# Quest markup for developing FAIR questionnaire modules for epidemiologic studies

**DOI:** 10.1186/s12911-023-02338-6

**Published:** 2023-10-25

**Authors:** Daniel E. Russ, Nicole M. Gerlanc, Brian Shen, Bhaumik Patel, Amy Berrington de González, Neal D. Freedman, Julie M. Cusack, Mia M. Gaudet, Montserrat García-Closas, Jonas S. Almeida

**Affiliations:** Division of Cancer Epidemiology and Genetics, National Cancer Institute, 9609 Medical Center Drive, Bethesda, MD 20892 USA

**Keywords:** Surveys and questionnaires, Data collection, Data commons, Data science, Epidemiologic methods

## Abstract

**Background:**

Online questionnaires are commonly used to collect information from participants in epidemiological studies. This requires building questionnaires using machine-readable formats that can be delivered to study participants using web-based technologies such as progressive web applications. However, the paucity of open-source markup standards with support for complex logic make collaborative development of web-based questionnaire modules difficult. This often prevents interoperability and reusability of questionnaire modules across epidemiological studies.

**Results:**

We developed an open-source markup language for presentation of questionnaire content and logic, Quest, within a real-time renderer that enables the user to test logic (e.g., skip patterns) and view the structure of data collection. We provide the Quest markup language, an in-browser markup rendering tool, questionnaire development tool and an example web application that embeds the renderer, developed for The Connect for Cancer Prevention Study.

**Conclusion:**

A markup language can specify both the content and logic of a questionnaire as plain text. Questionnaire markup, such as Quest, can become a standard format for storing questionnaires or sharing questionnaires across the web. Quest is a step towards generation of FAIR data in epidemiological studies by facilitating reusability of questionnaires and data interoperability using open-source tools.

**Supplementary Information:**

The online version contains supplementary material available at 10.1186/s12911-023-02338-6.

## Background

Questionnaires are key instruments for collecting information from participants in epidemiological studies. Moving from paper to web-based questionnaires can improve data quality and decrease the time and cost of questionnaire delivery and completion. [1] The development of web-based questionnaires can be facilitated by web applications that support complex logic and user-interface formatting, ideally following FAIR (Findable, Accessible, Interoperable, and Reusable) principles [2] so that the questionnaires are reusable and the data interoperable. Applications such as Survey Monkey or Google Forms allow researchers to develop questionnaires through proprietary user interfaces. However, these tools do not support complex logic. To support web-based administration, epidemiologists often use word processing software to develop annotated questionnaire versions that contain the static question text, dynamic text piped from responses, and logic that directs participants along a personalized path through the module. In addition, annotated documents may contain user-interface elements (e.g., introductory text, pop-up definitions, formatting) desired in the final product presented to the study participant. These documents are then used by programmers to develop web-based questionnaires, often using proprietary software that can handle the complex logic. Proprietary questionnaire platforms, such as Qualtrics, can handle complex questionnaires, but the entire software ecosystem is managed internally, which represents an impediment to the availability of questionnaire responses as data commons. [3] Clients program questions through a graphical interface occasionally adding custom code. While such platforms may share a question library with other paying users, they lack open-source representations that allow widespread reusability of questionnaires.

Ideally, the annotated questionnaire document would be in a human-readable, platform-independent, machine-readable, plain-text format with questions and logic that an application can render for the participant. Markup languages meet these requirements and can resemble the annotated documents produced by epidemiologists. Originally used to simplify development of HTML, markup has been used in many different contexts including writing books, software documentation, and within messaging applications. [4, 5] For questionnaires, describing the complex logic between questions and the interplay between questions and responses are easier to define using a markup language than using annotated word processing or spreadsheet documents. In addition, markup languages promote equitable research by providing free, open-source tools that enable reuse of questionnaires by scientists who may not be able to afford commercial tools, particularly those in low- and middle-income countries.

We developed the Quest open-source questionnaire markup and supporting applications in the public domain with the aim to remove barriers that prevent adoption of FAIR principles in epidemiology. [6] The major advancements that Quest brings are: a markdown format that allows reuse of questionnaire modules across studies; providing a default markdown renderer that integrates into a studies web application allowing studies to choose a backend system instead of forcing vender-specific backends; a standard markdown usable by commercial and open systems facilitating interoperability.

## Implementation

### Basic quest markup

In this section, we describe the basics of the markup. More complex markup is available, and a description can be found online in the Quest wiki. [7] In this section, italic text will be used to distinguish markup elements and their orchestration from the text describing them.

The basic markup structure for a questionnaire module is a series of questions. Questions are defined in the markup with a question id surrounded by square brackets followed by the question text and a set of responses. The first letter of a question id must be a capital letter (A-Z); the rest of the id can be capital letters, numbers, underscores, or octothorps (hash tags). Examples of valid question identifiers include *[Q]*, *[Q1]*, *[Q#1]*, and *[THIS_IS_VALID]*. The final question uses the question id [END].

#### Question syntax

As is the case for markup languages in general, the questions themselves are composed of plain text. The elements of each question are represented with simplified syntactic patterns mapped to another markup language, HTML. In essence, this follows the same rationale associated with other markup languages [4, 5]. Correspondingly, each question block consists of the question text and responses that can use a range of HTML form elements to handle different response types. The most common cases are *select one of the responses* or *select multiple responses*, which map into an HTML input type = radio or type = checkbox, respectively. The markup uses parentheses surrounding a value to represent a radio button and square brackets surrounding the return value for checkboxes. For the markup to remain consistent, return values cannot be valid question ids. A wide range of text and numeric input formats are supported, which are specified using a vertical bar followed by two underscores and another vertical bar, *|__|* for basic text values, while numeric input is specific by *|__|__|*. Other HTML element types can be specified using |*date*|, |*tel*|, and *|SSN|*. Quest’s GitHub wiki contains detailed information on additional response types, and a summary of current Quest markup is provided [see Supplementary Tables 1, Additional file 1]. Advanced developers can also have responsive grids that display multiple questions with the same responses. An in-browser application is provided at https://episphere.github.io/quest where the questionnaire markup can be tested interactively during development.

#### Questionnaire logic

The markup logic includes simple and more complex syntax to allow for skip logic. The simple logic is the arrow markup: *response -> question id*. The arrow indicates that if this *response* is selected, go to the question with the given id. This simple logic allows the developer to skip parts of the module that are not applicable to the participant. The no response markup, < *#NR -> question id*>, can be used for cases when the user does not select one of the responses.

The arrow markup adds a question to a stack (last in, first out list) of questions that assembled for the participant. When the stack empty, next question is assumed to immediately follow the current question. However, questionnaire modules may include follow-up questions for situations when the participant can choose multiple responses. In this situation, each response may contain arrows pointing to the follow up questions, and a default next question, in which *< -> question id >* points to the next question after all the follow-up questions are answered by the participant. All selected responses with arrows are added to the question list along with the default next question. The default next question is always added to the stack. Care should be taken with follow up questions. All additional follow-up questions must specifically be added to the stack with an arrow or the default arrow, otherwise they will be ignored as the next question will come from the stack regardless of where it appears in the markup.

Finally, for questions that cannot be skipped by any other means, a *displayif* mechanism can skip a question based on previous results. This basic syntax will cover the most straightforward situations, but there are situations where complex logic requires a more functional representation than conditional event algebra. That expert level logic grammar allows, for example, the definition of loops within the module. This is explained in Quest’s wiki [7] in detail.

**Fig. 1:**
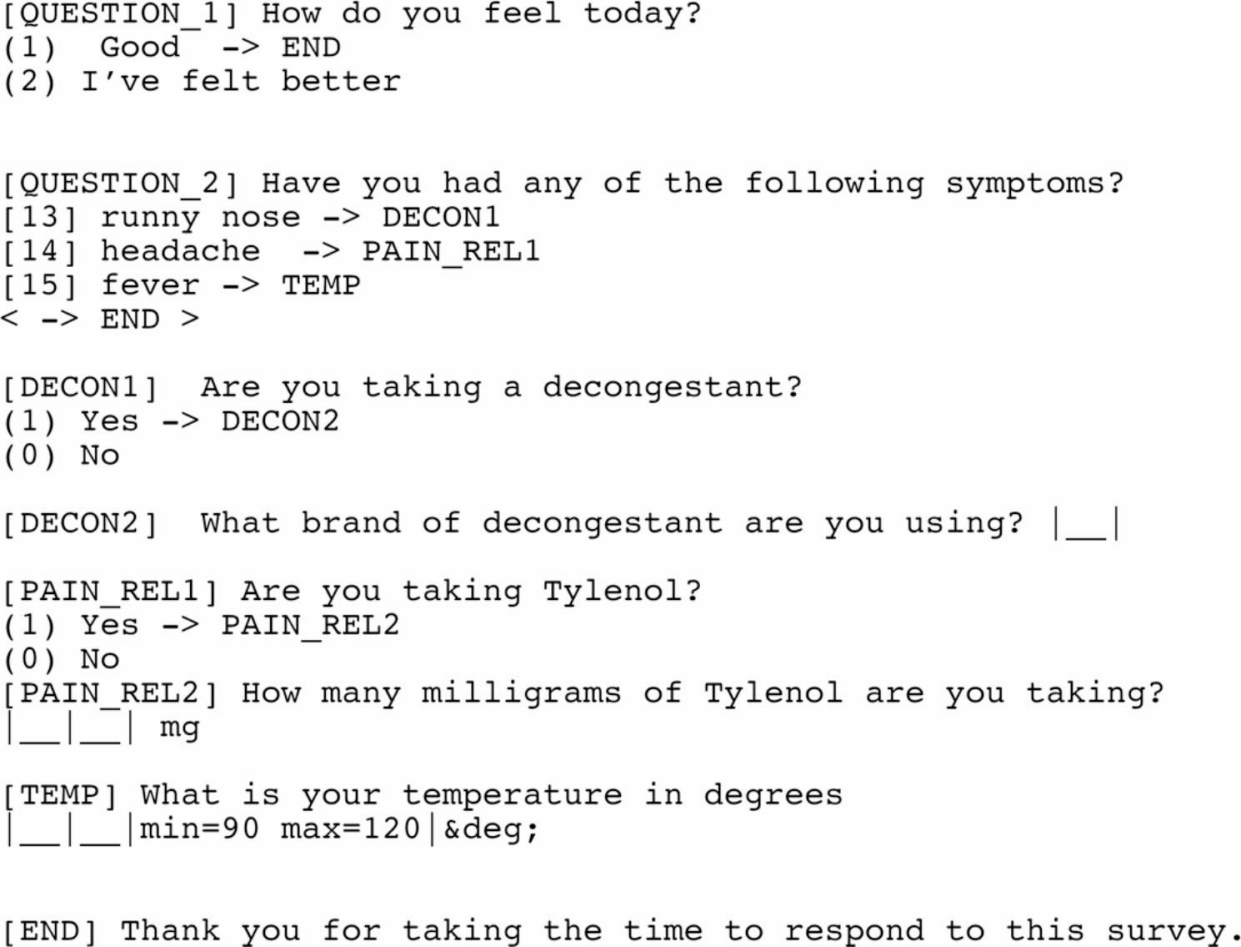
An example module of seven questions. The user is initially shown question QUESTION_1. If they select (1) then, they are immediately taken to question END, otherwise they are taken to QUESTION_2. In QUESTION 2, the user can select multiple answers. Assuming they select 13:runny nose and 15:fever, questions DECON1, TEMP, and END are added to a stack of questions to ask. In question DECON1, if the user selects (1) Yes then DECON2 is added immediately to the question stack, otherwise no questions are added, and the question stack is popped to select the question TEMP and then END. Notice that in the TEMP question a minimum of 90 and a maximum of 120 degrees are enforced. The JSON returned would look like: {QUESTION_1:2, QUESTION_2: [13,15], DECON1:0, TEMP:98}

Figure 1 is a simple example of a questionnaire module using Quest markup. This example illustrates both the markup and its rendering by the reference application: the markup is available online as a text file at https://danielruss.github.io/questionnaire/paper_example1.txt

### Supporting software

We developed a JavaScript library as an open-source reference implementation that renders the Quest markup into HTML for display in a browser. Working inside the browser provides access to all major computer operating systems, tablets, and smart phones. The library follows the module logic found in the markup displaying the appropriate question based on current and past responses. The quest library can be inserted into a standard HTML page or progressive web application using a content delivery network that caches code on GitHub (e.g. https://cdn.jsdelivr.net/gh/episphere/quest/replace2.js ). Our implementation caches the entire module in the browser DOM, and participant responses are saved in the browser’s indexedDB, an asynchronous NoSQL persistent storage native to the modern browser [8]. Participants with spotty or intermittent internet capabilities can therefore complete modules even if they lose the internet connection. Finally, results are transmitted back to the study via a callback function executed upon completion of the module. To support different studies with different preferences for data backend, Quest itself does not specify how or where studies store results. That is instead defined by the JavaScript callback function, which receive as the input argument a JSON object populated by the responses to the questionnaire.

We also provide an application for developing and presenting the Quest markup, which provides the developer the same view of the questions as the participant. As mentioned in the Methods section, the markup development tool is available at https://episphere.github.io/quest.

Styling the appearance and user interaction is a major component of the Quest renderer, which we’ve approached by independently parameterizing a Cascade Style Sheet document (CSS). Naturally, if no style is defined, for example rendering the questionnaire in a web application, the styling will be that of the application itself. This mimetic design implies that a cohort study using Quest will render questionnaires with the appearance of being native to the overall presentation configured for the Web Application.

## Results

**Fig. 2:**
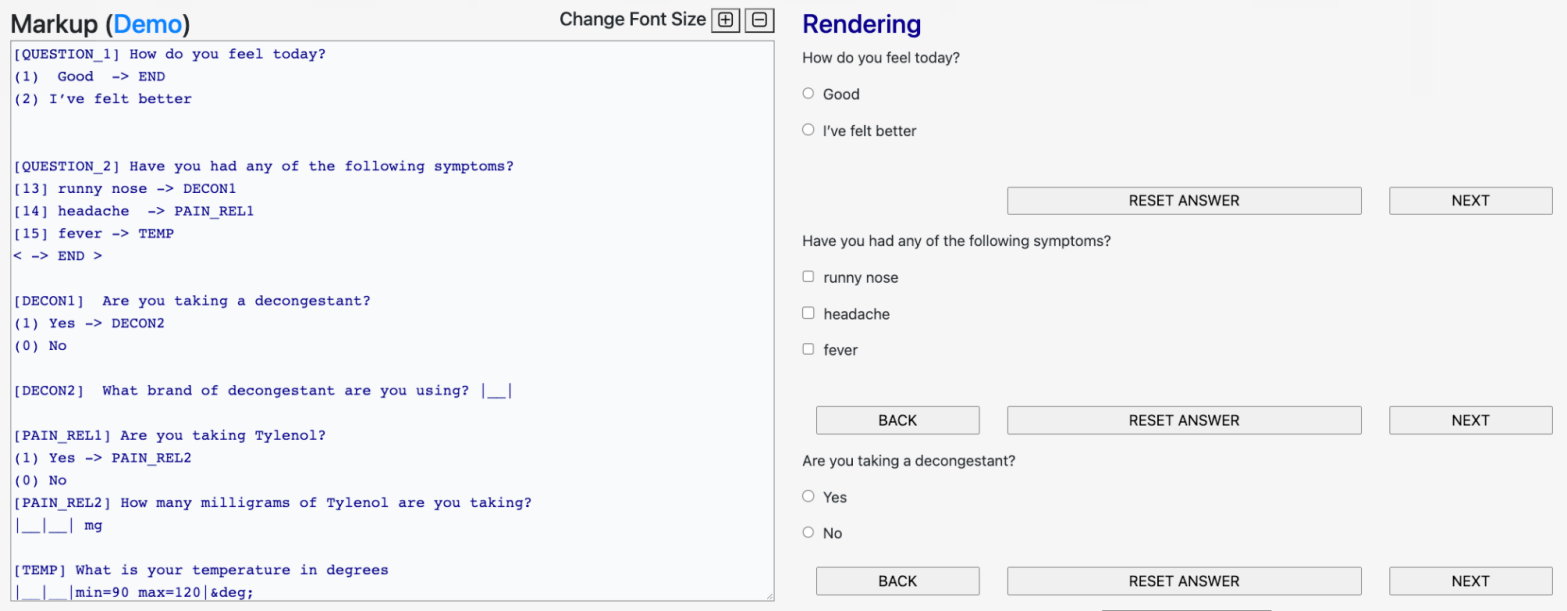
A screenshot of the live real-time composer/renderer showing a developer view of the module markup in Fig. 1 along with the unstyled, rendered HTML

Our JavaScript reference implementation markup development tool provides live editing/rendering of the Quest markup. Arguments are passed via the URL search parameters. For example, the markup is passed using the *url* parameter making the complete URL for markup from Fig. 1 https://episphere.github.io/quest?url=https://danielruss.github.io/questionnaire/paper_example1.txt and a screen shot is shown in Fig. 2. Logic and styling are activated by appending the URL of the style sheet to the *style* search parameters. A full screen participant view, useful for module and styling development, can also be triggered by adding the search parameter *run*.(e.g., https://episphere.github.io/quest/?style=Style1.css&run&url=https://danielruss.github.io/questionnaire/paper_example1.txt). An example of embedding the JavaScript markup renderer into a web application can be found at https://github.com/danielruss/AppUsingQuest.

**Fig. 3:**
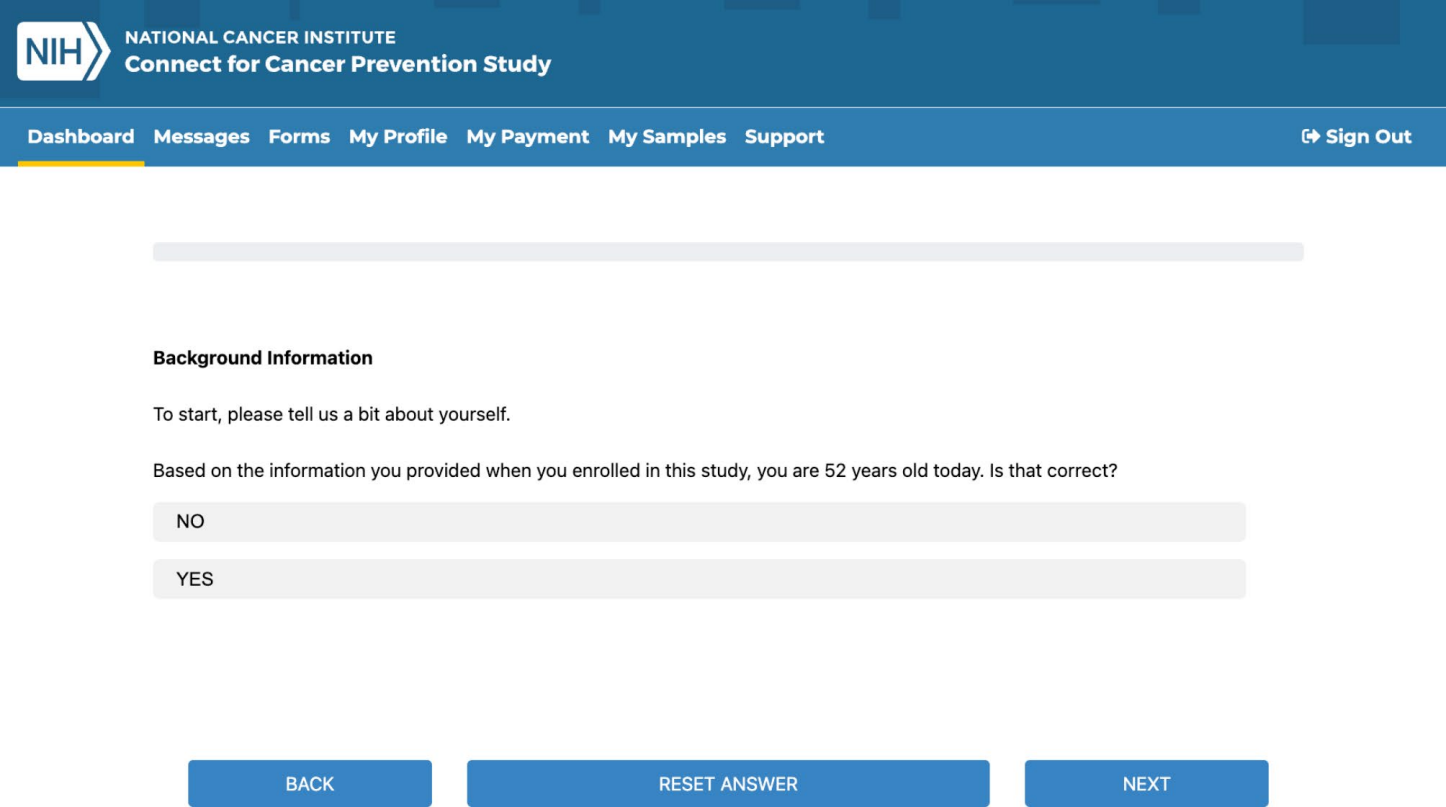
A screenshot of the *Connect for Cancer Prevention* progressive web application providing a questionnaire module. Notice that previous information, in this case age, can be provided to the module and displayed within a question

The first production use of the Quest markup and application was the *Connect for Cancer Prevention* study. [9] By calling the Quest JavaScript library, the study delivers multiple questionnaire modules into their participant progressive web application. Figure 3 is a screenshot of the *Connect* participant application displaying a question from a *Connect* questionnaire. Developers learned the Quest markup quickly by coding straightforward questions first, and then gradually learning more complex logic components such as those involving *displayif*, looping and *grid* logic.

## Discussion

We have developed a modular questionnaire markup language that defines a declarative formalism for specifying both the question content and the module logic. Quest markup is rendered in real-time into HTML. Epidemiologic studies can develop simple web-based applications, such as a PWA, that engage study participants and directly collect responses as a zero-footprint solution: no additional software is required to be installed to render a questionnaire formulated with the Quest markup language.

Currently, no open standard exists for interchange of questionnaires. In addition to markup, many other commonly used formats could have been chosen. We limited our discussion to human readable formats because we believe that benefits of easily understanding the format outweigh benefits of binary formats. With other formats, such as JSON or XML, the questionnaire programmer must carefully follow the recursive data format. A JSON brace in the wrong place or a misplaced XML end tag leads to bugs. Questionnaire programmer would need to convert every question into the format. In contrast, Quest markup mimics the appearance of the annotated documents provided by questionnaire developers. Furthermore, Quest markup is also a good interoperable standard because the human readable UTF-8 markup text lends itself to ready serializing to machine-friendly variable structures, such as JSON.

An additional major benefit of the combination of human-readable markup, privacy-preserving computation and browser-based development, is our ability to address FAIR principles. Specifically, Quest was designed as a testing ground for questionnaire commons addressing all the FAIR principles. The markup design exercise was put to the real-world test of making it work for the NCI/DCEG *Connect for Cancer Prevention S*tudy, which uses GitHub Pages to disseminate questionnaires with versioning and on the Web.

The guiding principles of FAIR are laid out in Box 2 of [2]. For findability, Quest accesses questionnaire modules via a persistent URLs acting as “globally unique and persistent identifier” [2] of for data. Metadata and indexing requirements for the modules can then be associated with these identifiers as linked data. Accessibility requires that the identifier be retrievable by “standard communication” using and “open, free, and universally implemented” protocol. [2] Quest then uses JavaScript to fetch the module needed to generate the corresponding HTML questionnaire rendering. If needed, applications using Quest can require authentication and authorization, for example, through external OAuth2 services along the interoperability standards gaining adoption for HL7-FHIR patient centric designs. [10] Interoperability is provided by the standard Quest markup itself, as the knowledge representation of questionnaires. Finally, reusability addresses attributes, licensing, provenance, and standards. Since the Quest renderer uses URLs as unique identifiers, updated versions of questionnaires receive new URLs documenting the historical lineage of marked up document.

In addition to the zero-footprint nature of web applications and the inherent privacy protection of operating client side, the reliance on web technologies to assemble in-browser applications brings with it an open-ended engineering platform. Specifically, additional client-side libraries can be integrated, as illustrated by the *Connect for Cancer Prevention* PWA calling the occupation coding service provided by SOCcer [11, 12]. The same extensibility is at hand for styling the questionnaire, by calling customized Cascading Style Sheets (CSS files). Notably, wearable and IoT devices are entering the communication ecosystem that surrounds study participants. Accordingly, online questionnaires follow advances in web technologies widening the range of participation models (e.g., voice, location, sensors, etc.).

Finally, Quest markup lays the foundation for other questionnaire rendering software. Allowing other teams to create more innovative, performant, and feature-rich online questionnaire software with a minimum shared set of expected features for epidemiological studies.

## Conclusion

Funding organizations, such as the NIH, increasingly expect grantees to make their data and software compliant with FAIR principles. [13] Accordingly, Quest markup language was developed to facilitate the collaborative development and maximize the reusability of questionnaire modules across multiple studies. As illustrated by the reference in-browser markup renderer, no specialized questionnaire servers are required. Neither to describe the questionnaire elements nor the logic underlying their presentation to cohort study participants. In a nutshell, Quest aims to enable the emergence of Questionnaire Commons that make the most of the nimble, extensive, and transparent nature of Web computing. To that end, Quest is provided with open source and in the public domain, with no restrictions on use or modification.

## Availability and requirements

Project name: Quest.

Project home page: https://github.com/episphere/quest.

Operating System(s): Platform independent.

Programming language: Quest markup and JavaScript.

Other requirements: Chrome based browser 108+, Firefox 108+.

License: MIT, U.S. federal employee – no copyright in US.

Any restriction to use by non-academics: no restrictions, please attribute work by citing this paper.

## Electronic supplementary material

Below is the link to the electronic supplementary material.

Supplementary Material 1

## Data Availability

Not Applicable.
