# Supplementary material for: Quest markup for developing FAIR questionnaire modules for epidemiologic studies
Source: BMC Med Inform Decis Mak. 2023 Oct 25;23:238. doi: 10.1186/s12911-023-02338-6 (PMC10598998; doi:10.1186/s12911-023-02338-6)
Supplement: Supplementary file 1 — Supplementary Material 1 [file 12911_2023_2338_MOESM1_ESM.docx]

Table 1. Elements of the Quest Markdown

| Markdown | Meaning |
| --- | --- |
| Markdown outside of a question | |
| [TEXT], [TEXT!], [TEXT?] | Square brackets where the first character is a capital letter [A-Z] followed by alphanumeric character or underscore [a-zA-Z0-9_] mark the start of a question. No spaces are allowed, and the text is the question id. (e.g., [Question_1]). Adding an exclamation point to the end of the question id requires the participant to answer the question to continue. Adding a question mark prompts the user to answer the question if no response is provided. |
| // | Comment everything beyond the two backslashes |
| Markdown inside a question | |
| #currentYear | Replaced with the current year |
| #currentMonth | Replaced with a numeric representation of the current month Jan=1, Feb=2, …, Dec=12 |
| #currentMonthStr | Replaced with a three letter representation of the current month Jan, Feb, … , Dec |
| (number) | Categorical response where only one option can be selected |
| [number], [number*] | Categorical response where one or more options can be shown. An asterisk means if this response is chosen, all other responses are clear. (e.g., None of the above, I prefer not to answer) |
| \|__\| \|___\| | When two underscores are used, one-line free text response (html input type=”text”). With three underscores, multi-line input (html text area) |
| \|__\|__\|id=TEXT min=mn max=mx\| | Numeric response with Id=TEXT, min value=mn, max value=mx |
| \|date\|id=TEXT\| | A date response with id TEXT |
| \|time\|id=TEXT\| | A time response with id TEXT |
| \|SSN\| | A Social Security number response |
| \|SSNsm\| | A 4-digit Social Security number response |
| \|@tel\| | A telephone number response |
| \|@\| | An e-mail address response |
| \|hidden\|id=ID\| | A hidden response |
| \|image\|URL\|height,width\| | An image available at URL with height and width |
| \|__\|__\|xor=XOR id=ID1\| | Exclusive OR. only allow one response from different responses that have an xor attribute with the same value. |
| response 🡪 TEXT | If response is chosen, add the question id to a list of questions the participant will be asked. |
| {$TEXT}  {$TEXT:default} | Pipe the response of question with id TEXT into the current question. Piped responses can take a default value that is returned when no response is found. |
| {$u:TEXT} | Pipes the response from variable input to quest from previous questionnaires. Must be passed in. |
| \|displayif=<condition>\|text and piped reponses\| | If the <condition> is true, print the text |
| [TEXT,displayif=<condition>] | If the <condition> is true, display this question |
| \|popup\|text\|Title\|popup-text\| | The “text” is printed, when the mouse goes over the word “text” a popup with title and popup text is shown |
| <loop> … </loop> | **ADVANCED:** Ask the questions in the loop multiple times until a condition breaks the loop |
| Functional Conditions (T/F unless mentioned) | |
| exists(id), doesNotExist(id) | Does a response exist for question with id? |
| allExist(…id), someExist(…id), noneExist(…id) | For an array of ids, do that all have responses, do some of them have responses, or do none of them have responses |
| valueOrDefaults(value,default) | Returns a value, if it does not exist, return the default |
| valueEquals(id,value) | Does the response for question with id equal the value |
| valueIsOneOf(id,…values) | Is the response for question with id one of an array of values. |
| valueIsBetween(id,…values) | Is the response for question with id between the upper limit |
| existingValues(<condition,value>,…) | Given a comma-separated list of conditions and values, return values when conditions are true. |
| selectionCount(Id) | Returns the number of selections made for question with Id |

Table 1. The elements of the Quest markdown are listed in the first columns along with descriptions of the meaning in the second column.
